# Foreign body aspiration in a rare tracheal anomaly: A case report

**DOI:** 10.1016/j.ijscr.2020.02.024

**Published:** 2020-02-15

**Authors:** Bassam Alghamdi, Salwa ALRashed ALHumaid, Talal Aljuhani, Fahad AlSaab

**Affiliations:** a Division of Otolaryngology–Head and Neck Surgery, Department of Surgery, King Abdulaziz Medical City and King Abdullah Specialist Children’s Hospital, Ministry of National Guard Health Affairs, Riyadh, Saudi Arabia; b Department of Pediatric Anesthesia, King Abdullah Specialist Children's Hospital, Ministry of National Guard Health Affairs, Riyadh, Saudi Arabia

**Keywords:** Tracheal bronchus, Foreign body aspiration, Rapid sequence intubation, Airway management, Intraoperative hypoxia

## Abstract

**Introduction:**

Tracheal bronchus is a rare anomaly in which an accessory bronchial branch originates superior to the tracheal bifurcation. It is usually incidentally found in patients with recurrent chest infection, persistent stridor and less commonly due to foreign body aspiration.

**Presentation of case:**

A 6-year-old medically and surgically free boy presented to the Emergency Department with severe shortness of breath. The patient was initially saturating well, but then suddenly worsened and was rushed for intubation. The patient was taken to the operating room for direct laryngobronchoscopy to retrieve the foreign body obstructing the airway. The foreign body was successfully retrieved, and the presence of a tracheal bronchus was confirmed.

**Discussion:**

The presence of tracheal bronchus has its implications on airway management.

**Conclusion:**

Knowledge about tracheal bronchus is of great importance to the otolaryngologist and anesthetist to maintain the airway.

## Highlights

• Tracheal bronchi are asymptomatic and require no medical nor surgical intervention.
• They may present with recurrent chest infections, stridor or may incidentally be found after foreign body aspiration.
• Awareness of the features of tracheal bronchus is crucial to maintain the airway and prevent any perioperative complications it may imply.
• Tracheal bronchus should be suspected in cases of atelectasis, hypoxemia, or both following endotracheal intubation.

## 1 Introduction

Tracheal bronchus is considered as a rare anomaly with an incidence ranging between 0.001%–2% [1]. It is usually found incidentally in patients with recurrent chest infections, persistent stridor or less commonly due to foreign body aspiration as in our case. Tracheobronchial foreign body aspiration is a true emergency in pediatric population, accounting for the majority of incidental deaths [2]. It is the leading cause of at-home accidental deaths in children less than 6 years of age [3]. Following foreign body aspiration, and with delay in diagnosis and hence in treatment, cardiopulmonary arrest and sudden death can follow [4].

In this case report, we present an incidental finding of a right accessory tracheal bronchus in a healthy 6-year-old child presenting to the Emergency Department (ED) with foreign body aspiration.

This project has been reported according to the SCARE criteria with guardian consent on behalf of the patient for publication purposes [5].

## 2 Case presentation

A medically and surgically free 6-year-old boy, with a weight of 22 kg and height of 122 cm, was brought to the ED of our hospital by his teacher with severe shortness of breath. The patient was witnessed ingesting popcorn when he suddenly started to develop cough and shortness of breath.

In the ED, the patient was agitated, drowsy, and semi-conscious. There was no obvious upper airway obstruction, but auscultation revealed absent air entry in the left lung with subcutaneous emphysema in the right side of the neck. His oxygen saturation was acceptable on oxygen supplementation.

**Fig. 1:**
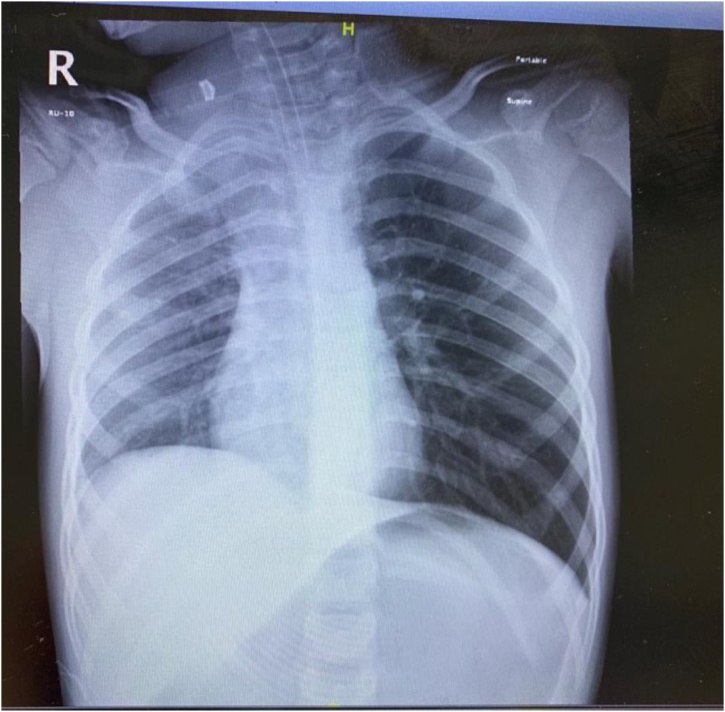
Chest x-ray after intubation showing hyperlucent left hemithorax with mediastinal shift to the contralateral side and flatting of ipsilateral hemidiaphragm.

Shortly after, patient became severely distressed and was intubated using midazolam, ketamine and succinylcholine. Chest x-ray was done after intubation and showed Endotracheal Tube (ETT) in good position, hyperlucent left hemithorax, flatting of ipsilateral hemidiaphragm, mediastinal shift to the right, and a radiopaque areain the left main bronchus (Fig. 1). Auscultation after intubation showed minimal flow in the left lung (improved compared to initial presentation) with some episodes of desaturation.

Otolaryngology – Head and Neck Surgery were contacted for urgent Direct Laryngoscopy and Bronchoscopy (DLB). After the patient was stabilized, he was taken to the operating room for DLB and foreign body removal with consent of the possible complications of bleeding, infection, inability to remove the foreign body, pneumothorax and/or teeth injury.

In the operating room, patient was intubated on bag mask ventilation. Air entry was diminished bilaterally with scattered wheezing in both sides. There was difficulty in bag mask ventilation with obvious expansion in the left side of the chest. The patient was connected to standard monitors. Initial end tidal CO_2_ was 104 mm Hg, arterial blood gas showed pH of 6.87, PaCO_2_ 181 mm Hg and PaO_2_ of 231 mm Hg.

General anesthesia was maintained with propofol infusion of 250 mcg/kg/min, and dexmedetomidine 1 mcg/kg/hr. One dose of dexamethasone 0.5 mg/kg was given to help in relieving the possible airway edema.

The patient was given succinylcholine during intubation in the ED followed by a dose of rocuronium, so the option of spontaneous ventilation was lost. The patient was maintaining his oxygen saturation (SaO_2_) on 100% O_2_ flow.

The decision was made to proceed with flexible fiberoptic scope through the ETT to delineate the anatomy.

First look was an unusual view of the foreign body which was seen saddling in the carina. The patient was extubated during flexible fiberoptic scope, so we proceeded with rigid bronchoscopy after irrigation with 2% lidocaine.

**Fig. 2:**
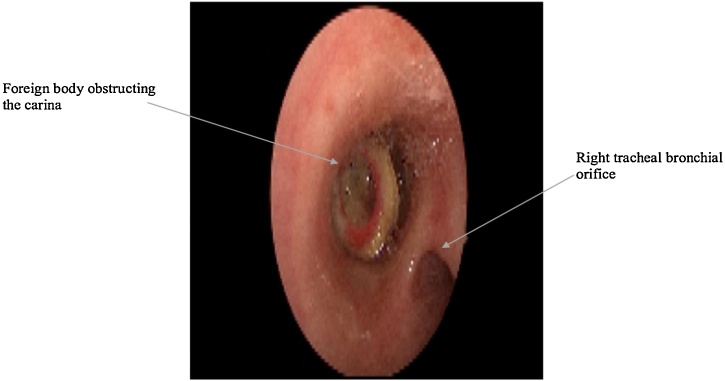
Foreign body obstructing the trachea at the level of tracheal bifurcation. A right tracheal bronchial orifice is seen.

**Fig. 3:**
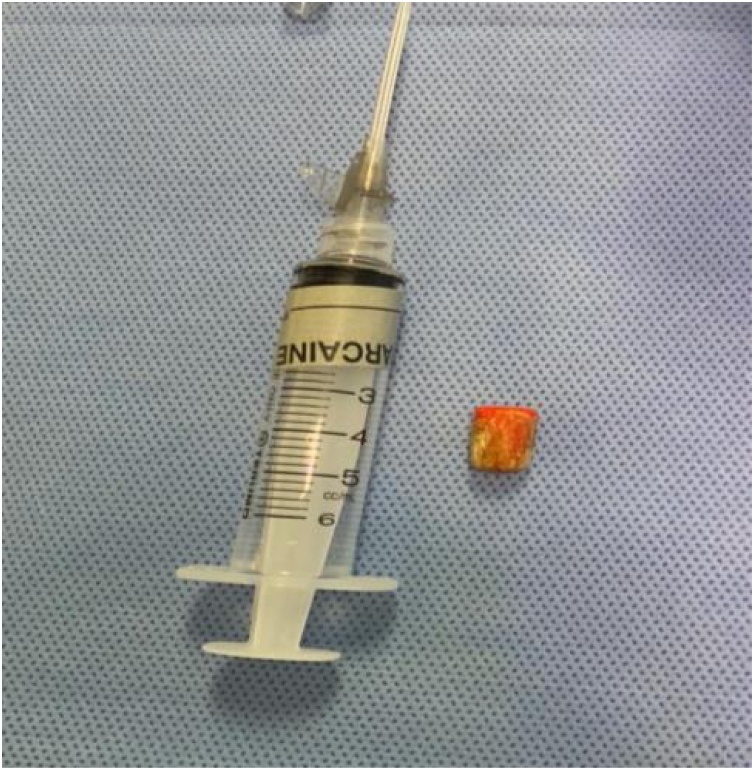
Foreign body was the eraser part of a pencil (no popcorn pieces).

**Fig. 4:**
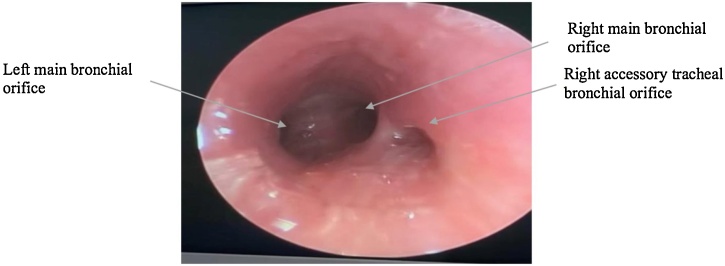
After removal of foreign body, re-examination revealed a clear airway with confirmed right tracheal bronchus.

While maintaining ventilation through the side port of the rigid bronchoscope, a foreign body was seen stuck in the trachea at the level of the carina, and a large right accessory tracheal bronchus was noted above the level of the foreign body (Fig. 2). The foreign body was successfully retrieved as one piece under vision using fiberoptic forceps (Fig. 3). A second look at the airway was done to exclude any other injuries and revealed a clear airway with no remaining foreign body and confirmed the presence of a right tracheal bronchus (Fig. 4).

**Fig. 5:**
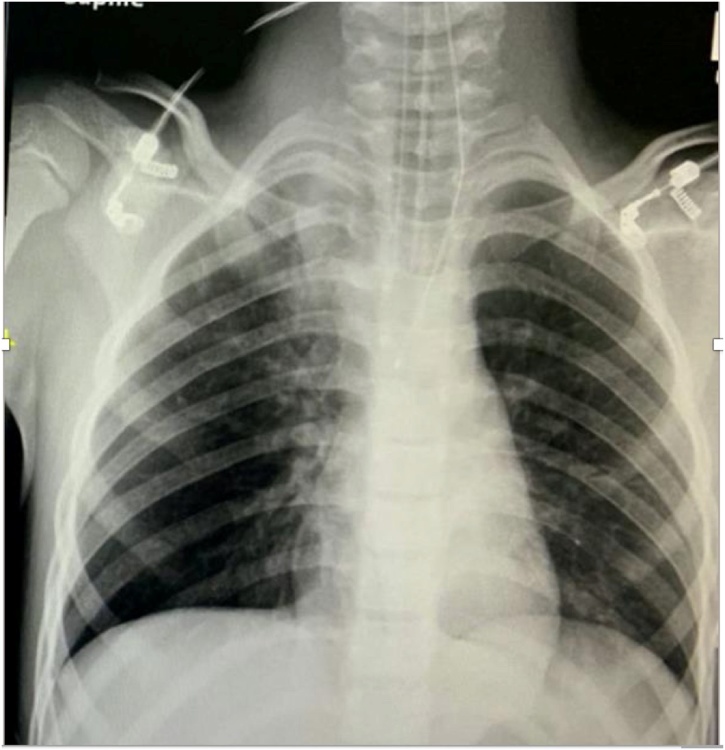
Repeated chest x-ray showing satisfactory ETT position and absence of pneumothorax.

After successful foreign body removal, another ETT was inserted and irrigation was done using normal saline. Airway entry improved, and arterial blood gas showed a pH of 6.95, PaCO_2_ of 141, and PaO_2_ of 40.3. Portable chest x-ray confirmed the ETT position and the absence of pneumothorax (Fig. 5). The patient was shifted from the operating room to the pediatric intensive care unit (PICU) fully sedated and intubated. The patient was monitored in PICU and was extubated the same day.

The patient was playful, tolerating orally, with no signs of respiratory distress and maintaining saturation on room air. He returned to his usual level of activity and was given dexamethasone 10 mg every 6 h (total of 4 doses). He was discharged home the following day in a good and stable condition with no need for further follow up.

## 3 Discussion

Initially described by Sandifort in 1785, a tracheal bronchus is a rare anomaly in which an accessory bronchial branch originates superior to the tracheal bifurcation with its incidence ranging between less than 0.001%–2% [1]. However, more recent literature in the late 20th century defines it as an ectopic bronchus that originates 2–6 cm above the carina [6]. A “pig bronchus” is when the entire right upper lobe bronchus arises from the trachea. It is rarely seen with a reported frequency of 0.2% [7]. Tracheal bronchi on the right side are more prevalent than left, with a prevalence of 0.1%–2% and 0.3%–1%, respectively [7]. This anomaly is most commonly asymptomatic; however, patients might present with recurrent chest infections, retained secretions, stridor, and to a lesser degree, foreign body aspiration.

Tracheal bronchus is usually detected during routine bronchoscopy for these indications [1,8,9,10,11]. The patient in our case had no history of recurrent chest infections nor upper airway obstruction.

Tracheal bronchus is classified into two types; displaced or supernumerary. The displaced type is when it is associated with an absence of a segmental branch in the upper lobe, thus representing a normal segmental bronchial division of the upper lobe of abnormal origin. The supernumerary type, on the other hand, is a true accessory bronchus associated with normal branching of the upper lobe bronchus [1].

Since tracheal bronchi are mostly asymptomatic, they are usually picked up incidentally as a radiographical finding [9]. Initial imaging, usually a chest x-ray, might demonstrate an accessory bronchus that arises from the supracarinal area, more commonly on the right side [12]. However, computed tomography has a higher diagnostic value and depicts the exact anatomy of the bronchopulmonary tree [12]. On the other hand, for a definitive view, bronchoscopy is used [13]. In our case, a rigid bronchoscope was used to retrieve the foreign body obstructing the airway at the level of the carina.

The presumed reason behind the patient’s ability to maintain saturation while paralyzed on positive pressure ventilation and in the presence of an obstructing foreign body; is the presence of a tracheal bronchus above the level of tracheal bifurcation permitting oxygen flow to the lobe aeriated by it.

A similar case to the one we present was reported in 1998 by Fourier et al., in which a 2-year-old girl presented to the ED with foreign body aspiration and signs of respiratory distress [14]. Chest x-ray revealed a moderately hyperinflated left hemithorax. Flexible bronchoscopy confirmed the presence of fragments of peanuts in the left main bronchus. Because of severe respiratory distress, she was intubated and ventilated, and transferred to PICU for rigid bronchoscopy to retrieve the foreign body. The ETT was positioned 2 cm above the carina. Rigid bronchoscopy was performed and showed the presence of a tracheal bronchus. Under general anesthesia, her SaO_2_ remained below 80%. Prior to foreign body retrieval, and with the placement of the rigid bronchoscope tip above the origin of the tracheal bronchus, a dramatic rise in oxygen saturation above 92% was noted. The fragments of the peanuts were removed uneventfully, and the patient required no further reintubation or oxygen administration after removal. Since the patient was otherwise asymptomatic, no further investigations were required.

A failure to recognize the presence of a tracheal bronchus during intubation may raise some problems. The side of the ETT tube might occlude the tracheal bronchus ultimately leading to atelectasis of the lobe aeriated by it. Also, theoretically, the tracheal bronchus can accidentally be intubated, which may result in pneumothorax or decreased aeriation in the remainder of the bronchopulmonary tree. Thus, a tracheal bronchus should be considered in the list of differential diagnoses for patients with unexplained intraoperative hypoxemia [15,16].

Treatment of tracheal bronchus depends on the severity of symptoms it causes. In most cases, tracheal bronchus is asymptomatic, and no surgical or medical intervention is required. However, in cases where a tracheal bronchus causes recurrent respiratory infections, resection of the accessory bronchus along with the lobe it supplies is the mainstay of treatment to avoid further pulmonary damage [15].

## 4 Conclusion

Most tracheal bronchi are asymptomatic and require no medical nor surgical intervention. However, they may present with recurrent chest infections, stridor or may incidentally be found after foreign body aspiration as in our case. Knowledge about tracheal bronchus is of great importance to the otolaryngologist and anesthetist to maintain the airway and prevent the unanticipated perioperative complications it may imply. Tracheal bronchus should be suspected in cases of atelectasis, hypoxemia, or both following endotracheal intubation.

## Sources of funding

No source of sponsorship.

## Ethical approval

Research has been approved by the Research Ethical Committee of King Abdullah International Medical Research Center.

## Consent

Consent was taken from guardian on behalf of the patient for publication purposes.

## Author contribution

Salwa AlRashed ALHumaid: Data collection, data analysis & interpretation and writing the paper.

Bassam Alghamdi: Data collection, data analysis & interpretation and writing the paper.

Fahad Alsaab: Study concept & design, data collection, data analysis or interpretation, writing the paper.

Talal Aljuhani: Data analysis & interpretation and writing the paper.

## Ethical approval

Research has been approved by the Research Ethical Committee of King Abdullah International Medical Research Center.

## Registration of research studies

Non Applicable.

## Guarantor

Fahad Alsaab.

Salwa ALRashed ALHumaid.

## Provenance and peer review

Not commissioned, externally peer-reviewed.

## Declaration of Competing Interest

No conflicts of interest.
